# A new species of *Malletia* (Bivalvia, Malletiidae) and new records of deep-water bivalves from Pacific Southern Colombia

**DOI:** 10.3897/zookeys.762.20335

**Published:** 2018-05-30

**Authors:** Nancy Yolimar Suárez-Mozo, Adriana Gracia, Paul Valentich-Scott

**Affiliations:** 1 Posgrado en Ciencias del Mar y Limnología, Universidad Nacional Autónoma de México – UNAM, Mazatlán, Sinaloa, México Posgrado en Ciencias del Mar y Limnología–Universidad Nacional Autónoma de México– UNAMMazatlanMexico; 2 Programa de Biología, Universidad del Atlántico, Km 7 Antigua Vía Puerto Colombia, Atlántico, Colombia Programa de Biología – Universidad del Atlántico, Km 7 Antigua Vía Puerto Colombia, Atlántico ColombiaBarranquillaColombia; 3 Instituto de Investigaciones Marinas y Costeras – Invemar, Santa Marta, Colombia Instituto de Investigaciones Marinas y Costeras–Invemar. Santa Marta, ColombiaSanta MartaColombia; 4 Department of Invertebrate Zoology, Santa Barbara Museum of Natural History, 2559 Puesta del Sol, Santa Barbara, CA 93105 USA Santa Barbara Museum of Natural HistorySanta BarbaraUnited States of America

**Keywords:** Bivalvia, benthos, Colombia, deep-water, *
Malletia
*, Malletiidae, Tumaco

## Abstract

In order to enhance the understanding of Pacific Colombia’s deep-water marine fauna, a benthic research cruise (2012 TUM Offshore 6 and 7) was conducted off the coast of the Department of Nariño, in southern Colombia. Biological, oceanographic and sediment samples from the continental shelf and slope were collected at depths between 350 and 941 m. A new species of *Malletia* obtained on that cruise is described and compared with other species from the eastern Pacific. Sixteen species of bivalve mollusks (belonging to 12 families and 15 genera) were identified. Five of them were the first records for Pacific Colombia (*Jupiterialobula*, *Limatulasaturna*, *Lucinomaheroica*, *Cuspidariapanamensis*, and *Dallicordiaalaskana*). Four of them had geographic distributions that now extend to Tumaco at the southern end of Nariño.

## Introduction

Throughout the past decade, the search for hydrocarbon and natural gas reserves in Colombia (Pacific and Caribbean coast) has sparked an interest in the country’s remote deep-sea regions. This has resulted in intensified deep-sea baseline studies, primarily along the continental shelves and slopes. Nevertheless, deep-sea studies face logistical and cost limitations, including the availability of research vessels and proper equipment for collecting samples.

Despite the increase in knowledge during recent years, the presently known range of many invertebrates groups inhabiting soft sediments, including mollusks, is still fairly fragmentary in remotes parts of the Colombian Pacific. There is a lack of published data on the biology, functional morphology, ecology, development and dispersal mechanisms for these invertebrates, as well as a lack of baseline faunal inventories. Thus, the true biodiversity of the Pacific Colombian deep-sea must be vastly underestimated.

As a result of recent Colombian expeditions, a rich benthic fauna inhabiting of the deep-seas of Pacific Southern Colombia has been discovered, but few species of mollusks have been described when compared with the mollusks from the coasts of the Colombian Atlantic (e.g., Ardila-Espitia and Diaz 2002, Simone and Gracia 2006, Gracia and Ardila-Espitia 2009).

In the context of faunal inventories, the tropical west coast of America is well documented, with 890 species of bivalves presently recorded (Coan and Valentich-Scott 2012). For northwestern South America to Peru, a basic knowledge of deep-sea bivalve mollusks has been covered by a few recent publications (e.g., Gracia and Valentich-Scott 2014; Paredes et al. 2016). The investigations in Pacific Colombian waters have hitherto focused mainly on the coastal zones (e.g., Cantera et al. 1979, Cosel 1984, Díaz et al. 1997, Cantera 2010, López de Mesa and Cantera 2015) rather than zones farther offshore (e.g., Hertlein and Strong 1955). Gracia and Valentich-Scott (2014) documented the bivalves off the Department of Choco (Colombian North Pacific) where more than 38 species of bivalves were found, 34 of which were new records for the country.

The current work presents a systematic and annotated list of bivalve species collected in the southern Colombian Pacific region. Each entry includes the species’ geographic and bathymetric distribution, plus additional remarks and observations. From the above, several species stand out as being first records for the country. We are also including the description of a new species uncovered in this survey. Our records represent a significant expansion in the knowledge of the Pacific Colombian bivalve fauna, but much more sampling and analysis is needed when one takes into account the large geographic extent of this region.

## Materials and methods

### Study area

**Figure 1.**
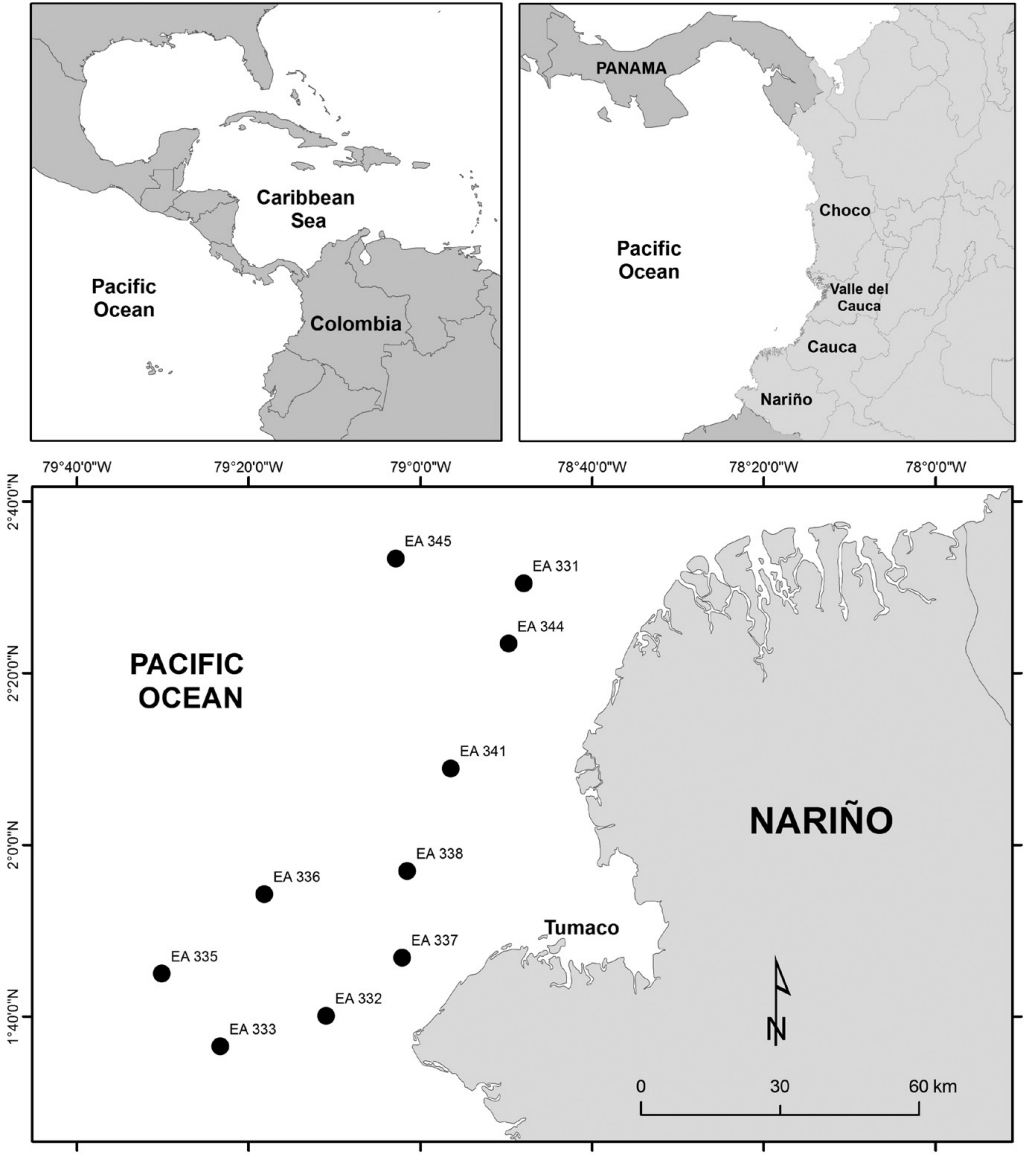
Locations along the Colombian Pacific Ocean where bivalves were collected.

The present study was carried out in the tropical eastern Pacific Ocean (Fig. 1). The study area (TUM Offshore Blocks 6 and 7) covered 7,308 km^2^ and extended from Sanquianga National Nature Park in the Department of Nariño to the Colombia-Ecuador border. The region is influenced by continental contributions from Tumaco Bay, as well as by numerous rivers, including the Patia and Mira (IDEAM et al. 2007). This study area is part of the research project known as the “Biological and physical baseline survey of TUM Offshore Blocks 6 and 7 subject to hydrocarbon exploration” (ANH-Invermar 2013).

### Sample collection

Samples were collected from 4–22 December 2012, on board the fishing vessel *Perla Verde*. Collection depths ranged from 350–941 m. All the 13 trawls made during the survey were taken in soft and homogenous sea beds. Ten of the 13 sampling locations included bivalves.

Each sample was collected with a benthic semi-balloon trawl net (9 × 1 m) for 10 minutes at a speed of 3 knots. Because the exact time at which the net opened was unknown, sampling was semi-quantitative. We acknowledge that this sampling technique could have missed small and microscopic species as would be taken by epibenthic sleds, but the equipment needed for this method was not available to us. Collected material was coarsely sorted on deck and later identified to lower levels at the Museo de Historia Natural Marina de Colombia (MHNMC) which is part of Instituto de Investigaciones Marinas y Costeras (INVEMAR). The empty valves were air-dried, while the soft-bodied organisms were preserved in 70% ethanol.

Specimen identification was based upon shell characters. Museum materials, bibliographic references and bivalve taxonomic experts were consulted to confirm the results (e.g., Dall 1896, 1908; Keen 1971; Coan and Valentich-Scott 2012). The identified material included many complete living organisms as well as empty shells of bivalves. The systematic order of this list corresponds to that proposed by Coan and Valentich-Scott (2012). Specimens from this study, including other mollusks not analyzed in this work (e.g., gastropods, chitons, and cephalopods), now reside at the MHNMC’s mollusk collection in Santa Marta, Colombia.

Oceanographic data were collected with an Idronaut CTDO marine profiler (yielding data for conductivity, temperature, depth, and oxygen concentration) at sites S333 and S334, both of which contained bivalves (Table 1). Sediment core sets were collected five sites (S331, S333, S334, S341, and S345) with a Gomex II Box corer that had a 32 liter storage capacity. Sediment grain analysis revealed a predominance of silt (Table 1). Grain size classification was conducted according to Folk (1974). All samples were classified as silts; 57% of samples were purely silts, while the remaining 43% also contained sand and gravel fractions (INVEMAR-ANH 2013).

**Table 1.** Size distribution of analyzed sediment samples according to INVEMAR-ANH (2013).

Station	Depth (m)	% Gravel	% Sand	% Silt
331	320	0.1	23.2	76.7
333	833	0.0	1.0	99.0
334	864	0.0	0.7	99.3
341	894	0.2	1.1	98.7
345	570	0.0	49.7	50.3

### Abbreviations

**EA** Trawl station; S sediment station

**MHNMC** (Spanish acronym) Museo de Historia Natural Marina de Colombia

**TUM OFF** Tumaco-Offshore

## Results

A total of 324 bivalve specimens was collected, including 247 empty or disjointed valves and 77 live-collected organisms. The specimens were sorted into 16 species, 15 genera, and 12 families; five species were new observations in the Colombian Pacific. The known geographic range of several species has now been expanded to the Department of Nariño.

Below is included a listing of the species collected, station data, live-dead status for each specimen, remarks on new verified localities, previously reported distributions for the species, plus general remarks. We have also included an illustration for all newly documented species in Colombia i.e., those other than *Nuculaiphigenia*, *Orthoyoldiapanamensis* and *Delectopectenzacae* which were previously reported for the Pacific of Colombia by Gracia and Valentich-Scott (2014).

### Systematics

### Class BIVALVIA Linnaeus, 1758

#### Subclass PROTOBRANCHIA Pelseneer, 1889

##### Order NUCULIDA Dall, 1889

###### Superfamily NUCULOIDEA J.E. Gray, 1824

####### Family NUCULIDAE J.E. Gray, 1824

######## Genus *Ennucula* Iredale, 1931

######### Ennucula panamina

Taxon classificationAnimaliaNuculidaNuculidae

(Dall, 1908)

Fig. 2

########## Examined material.

1 valve plus 1 live specimen EA 336 (1.9045°N, 79.3030°W) at 612 m (INV MOL9797, INV MOL9796), 1 live specimen EA344 (2.3905°N, 78.8288°W) at 656 m (INV MOL9796), plus 1 live specimen EA 335 (1.7499°N, 79.50177°W) at 866 m (INV MOL9799).

**Figure 2–12.**
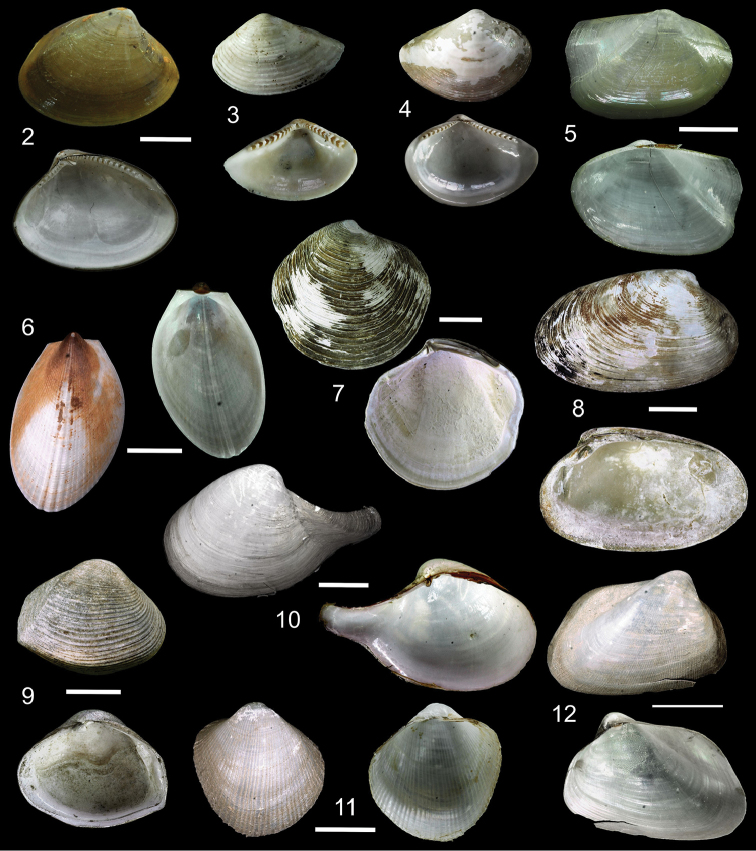
**2** Exterior and interior views of *Ennuculapanamina***3***Jupiterialobula* (total length = 4.2 mm) **4**Neilonellacf.atossa (total length = 5 mm) **5***Malletiagoniura***6**Limatulacf.saturna**7***Lucinomaheroica***8**Calyptogenacf.gallardoi**9***Carycorbula* sp. **10***Cuspidariapanamensis***11***Dallicordiaalaskana***12**Lyonsiellacf.magnifica. Scale bars: **2, 5, 6, 9, 11** 5 mm. **7, 8, 10, 12** 10 mm.

########## New location.

Off Nariño, Colombian Pacific.

########## Distribution.

Panama to Peru (Coan and Valentich-Scott 2012).

########## Remarks.

New species record for the Colombian Pacific.

######## Genus *Nucula* Lamarck, 1799

######### Subgenus Lamellinucula Schenck, 1944

########## Nucula (Lamellinucula) iphigenia

Taxon classificationAnimaliaNuculidaNuculidae

Dall, 1896

########### Examined material.

1 valve plus 2 live specimens EA331 (2.5078°N, 78.7993°W) at 350 m (INV MOL9794, INV MOL9795).

########### New location.

Off Nariño, Colombian Pacific.

########### Distribution.

Panama to Peru (Coan and Valentich-Scott 2012), Choco-Colombia (Gracia and Valentich-Scott 2014).

########### Remarks.

Previously encountered in Colombia in the Department of Choco at a depth of 300 m (Gracia and Valentich-Scott 2014).

##### Order NUCULANOIDA D.C. Carter & M.R. Campbell, 2000

###### Superfamily NUCULANOIDEA H. & A. Adams, 1858

####### Family NUCULANIDAE H & A. Adams, 1858

######## Genus *Jupiteria* Bellardi, 1875

######### Jupiteria lobula

Taxon classificationAnimaliaNuculanoidaNuculanidae

(Dall, 1890)

Fig. 3

########## Examined material.

2 valves EA337 (1.7811°N, 79.0351°W) at 530 m (INV MOL9791), 2 valves EA331 (2.5078°N, 78.7993°W) at 350 m (INV MOL9792).

########## New location.

Colombian Pacific.

########## Distribution.

Mexico to El Salvador (Coan and Valentich-Scott 2012).

########## Remarks.

These records represent a new southern limit for this species. All the specimens were small (approx. 4 mm), but they were nearly identical to small specimens of *Jupiterialobula* from Mexico and also the type specimens. The presence of dead valves at different stations and the distance from previous records suggest that this species is living in Colombia.

####### Family NEILONELLIDAE Schileyko, 1989

######## Genus *Neilonella* Dall, 1881

######### Neilonella cf. atossa

Taxon classificationAnimaliaNuculanoidaNeilonellidae

(Dall, 1908)

Fig. 4

########## Examined Material.

2 valves EA337 (1.7811°N, 79.0351°W) at 530 m (INV MOL9793).

########## New location.

Off Nariño, Colombian Pacific.

########## Remarks.

The identity of this species cannot be confirmed without a detailed comparative examination of additional material. It is potentially a new species.

####### Family MALLETIIDAE H. & A. Adams, 1858

######## Genus *Malletia* des Moulins, 1832

######### Malletia goniura

Taxon classificationAnimaliaNuculanoidaMalletiidae

Dall, 1890

Fig. 5

########## Examined material.

8 valves EA341 (2.1484°N, 78.9409°W) at 934 m (INV MOL9774), 7 live specimens EA341 at 934 m (INV MOL9775), 3 valves EA335 (1.7499°N, 79.5017°W) at 855 m (INV MOL9776), 6 live specimens EA335 at 866 m (INV MOL9777), 2 valves EA333 (1.6087°N, 79.3883°W) at 836 m (INV M9778), 4 live specimens EA333 at 836 m (INV MOL9779), 3 valves EA338 (1.9490°N, 79.0257°W) at 941 m (INV MOL9780).

########## New location.

Off Nariño, Colombian Pacific.

########## Distribution.

Panama to Peru (Coan and Valentich-Scott 2012).

########## Remarks.

These specimens represent the shallowest bathymetric records so far for *Malletiagoniura* (836–941 m). It has previously been collected in deeper waters (1,500–3,300 m depth) (Coan and Valentich-Scott 2012).

######### Malletia tumaquensis sp. n.

Taxon classificationAnimaliaNuculanoidaMalletiidae

http://zoobank.org/5DB5C232-C972-4E67-914D-AECB44A92CD8

Figs 17–20 , 22

########## Description.

**Shell shape**: Shell equivalve, subquadrate, moderately inflated, thin, gaping at ends, longer than high (length to height ratio 1:0.5), inequilateral, much longer posteriorly. Umbones moderate in size, located about one-third of shell length from anterior end. Lunule broad, shallow, weakly outlined, raised medially. Escutcheon absent. Anterodorsal margin angled ventrally from umbo; posterodorsal margin straight from umbo. Anterior end narrowly rounded, posterior end truncate. Strong radial keel extending from umbo to posterior margin, with deep radial sulcus immediately dorsal to it. Left valve with low radial undulations extending from near umbone to posteroventral margins, right valve with little or no undulation. Anteroventral and posteroventral region slightly undulate. Inner ventral margin smooth. Interior of valves smooth and porcelaneous.

**Adductor muscle scar and pallial scars**: Pallial line weakly impressed; pallial sinus broad, shallow. Adductor muscle scars subequal, subovate and moderate in size.

**Sculpture and periostracum**: Exterior sculpture of fine commarginal striae. Periostracum thin, adherent, glossy, pale yellow to light brown, often with commarginal color bands.

**Hinge**: Hinge with 2 distinct series of teeth without any separation between them; approx. 12 anterior teeth, larger than posterior teeth; approx. 39–52 posterior teeth. Ligament external, sunken, opisthodetic, narrow, dark brown, extending nearly 3/4 the length of posterodorsal margin.

**Anatomy**: Foot large, deeply cleft medially, wide at neck; labial palp and palp proboscis anterior; labial palp large, with 2 distinct regions with finer and heavier lamellae; palp proboscis very long, coiled.

########## Material Type.

**Holotype**: INV MOL9782; paired valves with soft body, length 33.2 mm, height 16.4 mm, width 11.8 mm.

########## Paratypes.

See Table 2 for measurements and length/height dimensions.

**Table 2.** Measurements of type specimens of *Malletiatumaquensis* sp. n.

Specimen	Length (mm)	Height (mm)	Width (mm)	Length/height
**Holotype INV MOL9782**	33.2	16.4	11.8	2.0
**Paratype 1 INV MOL1161**	30.5	15.7	11.4	1.9
**Paratype 1 INV MOL1161**	32.7	16.6	11.1	1.9
**Paratype 1 INV MOL1161**	30.4	15.1	10.3	2.0
**Paratype 2 INV MOL1162**	28.7	14.2	9.7	2.0
**Paratype 2 INV MOL1162**	28.4	14.7	9.8	1.9
**Paratype 2 INV MOL1162**	26.5	14.3	9.0	1.8
**Paratype 3 INV MOL1163**	26.1	13.4	9.0	1.9
**Paratype 3 INV MOL1163**	24.9	13.1	9.0	1.9
**Paratype 3 INV MOL1163**	27.2	13.3	9.6	2.0

########## Type locality.

Colombia, Nariño, off Tumaco Bay. St. EA337 (1.7811°N, 79.0351°W); depth 530 m. Collected November 2012.

########## Habitat.

Soft bottom.

########## Additional (non-type) material.

75 valves EA337 at 530 m (INV MOL9781) plus 19 live specimens EA337 at 530 m (INV MOL9782).

########## Distribution.

The species is currently only known only from the type locality.

########## Etymology.

This species is named in honor of the municipality of Tumaco, Nariño, where this study was conducted.

########## Differential diagnosis.

*Malletiatumaquensis* sp. n. is similar in shape to *M.alata* Bernard, 1989. However, consistent differences exist in conchological features (i.e., *M.tumaquensis* is more elongate, while *M.alata* has an alate process) and anatomical characteristics (i.e., very long, thin palp proboscis in *M.tumaquensis*) makes it a readily distinguishable new species. Ecologically, *M.tumaquensis* has a shallower depth distribution (530 m) than that of *M.alata* (740 m, Coan and Valentich-Scott 2012). Table 3 summarizes the shell characteristics of all the *Malletia* species recorded in the eastern Pacific Ocean.

**Table 3.** Summary of shell characters of *Malletia* species from the Pacific Ocean (after Coan and Valentich-Scott 2012).

Species	Shape	Type locality	Reported depth range (m)	Maximum Length (mm)	Posterior end	Hinge
*Malletiaalata* F. R. Bernard, 1989	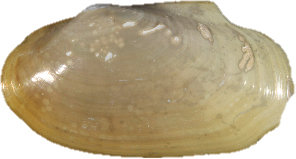	San Diego Trough, California, USA	1200	30	Straight, forming alate process	About 11–13 anterior teeth; about 45 posterior teeth
*Malletiaarciformis* Dall, 1908	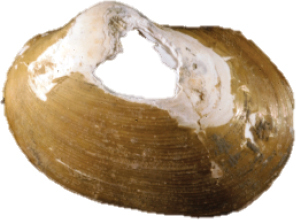	Off Acapulco, Guerrero, Mexico	902	11	Broadly flared, rounded	10–13 anterior teeth; 13–17 posterior teeth
*Malletiabenthima* Dall, 1908	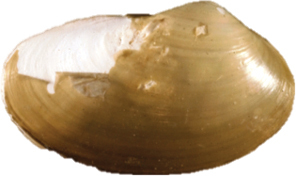	Off Acapulco, Guerrero, Mexico	902	10	Produced, broadly rounded	12–13 anterior teeth; 13–17 posterior teeth
*Malletiafaba* Dall, 1897	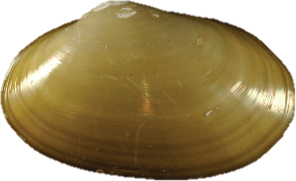	Off Queen Charlotte Islands, British Columbia, Canada	200–1600	10	Broadly rounded	About 9 anterior teeth; about 32 posterior teeth
*Malletiagoniura* Dall, 1890	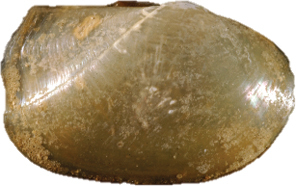	Gulf of Panama, Panama	1500–3300	13	Flaring dorsally, truncate	14–19 anterior teeth; 27–30 posterior teeth
*Malletiaperuviana* Dall, 1908	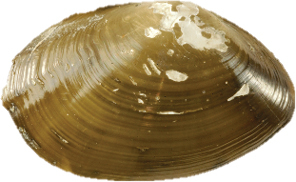	Off Punta Aguja, Piura, Peru	1900	28	Broadly rounded	10–11 anterior teeth; 33–36 posterior teeth
*Malletiatruncata* Dall, 1908	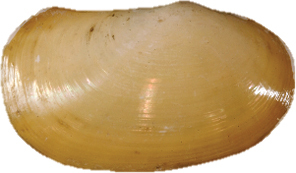	Cascadia Plain, Oregon, USA	2700–4134	30	Flaring, compressed, truncate	About 18 anterior teeth; about 30 posterior teeth
*Malletiatumaquensis* sp. n.	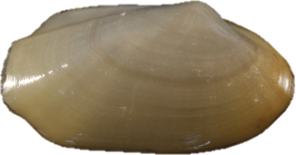	Off Tumaco Bay, Nariño, Colombia	530	33	Truncate	12 anterior teeth; 39–52 posterior teeth

########## Remarks.

Members of the family Malletiidae occur throughout the Pacific and Atlantic Oceans with most records from deep-water (Coan and Valentich-Scott 2012, Kamenev 2015). *Malletiatumaquensis* is distinguished from the seven other species occurring in tropical west America by its more subquadrate and longer shell. Including our record, this represents the third species of the genus reported for the Colombian Pacific (i.e., *M.tumaquensis*, *M.truncata* and *M.goniura*).

**Figure 13–20.**
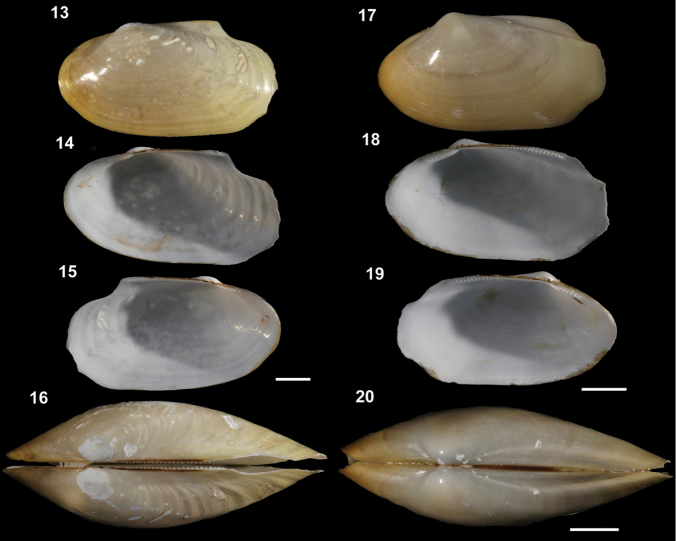
**13–16***Malletiaalata* (Holotype LACM 2343) **13** External view **14–15** Internal view of the shell, right and left respectively **16** Dorsal view (total length = 30 mm) **17–20***Malletiatumaquensis* sp. n. **17** External view **18–19** Internal view of the shell, right and left valves, respectively **20** Dorsal view. Scale bars: 0.5 mm.

**Figure 21–22.**
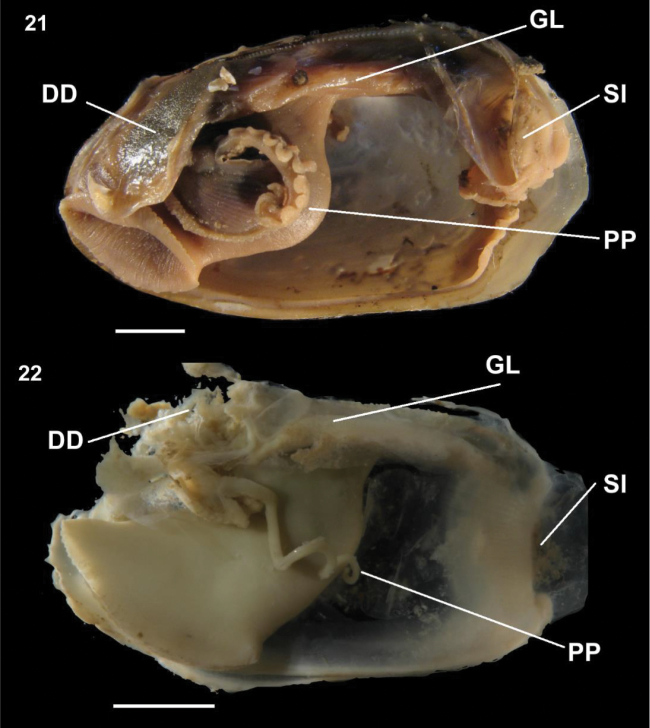
**21***Malletiaalata* (EMU7054) Gulf of California, Mexico, lateral view of anatomy **22***Malletiatumaquensis* sp. n. Lateral view of anatomy. Scale bars: 0.5 mm. Abbreviations: **DD** Digestive diverticula, **FT** Foot, **GL** Gill, **SI** Siphon, **PP** Palp proboscis.

####### Family YOLDIIDAE Dall, 1908

######## Subfamily YOLDIINAE Dall, 1908

######### Genus *Orthoyoldia* Verrill & Bush, 1897

########## Orthoyoldia panamensis

Taxon classificationAnimaliaNuculanoidaYoldiidae

(Dall, 1908)

########### Examined material.

10 valves EA344 (2.3905°N, 78.8288°W) at 656 m (INV MOL9812), 6 live specimens EA344 at 656 m (INV MOL9813), 5 valves EA337 (1.7811°N, 79.035139° W) at 530 m (INV MOL9814), 4 valves EA337 at 530 m (INV MOL9815), 4 live specimens EA332 (1.6677°N, 79.1826°W) at 730 m (INV MOL9816), 2 live specimens EA345 (2.5557°N, 79.0476°W) at 668 m (INV MOL9817).

########### New location.

Off Nariño, Colombian Pacific.

########### Distribution.

Mexico to Peru (Coan and Valentich-Scott 2012), Choco-Colombia (Gracia and Valentich-Scott 2014).

########### Remarks.

*Orthoyoldiapanamensis* has previously been collected in depths from 120 to 475 m in Colombia (Gracia and Valentich-Scott 2014). This study extends the bathymetric range to 730 m in the Colombian Pacific.

##### Order PECTINIDA J.E. Gray, 1854

###### Superfamily PECTINOIDEA Rafinesque, 1815

####### Family PECTINIDAE Rafinesque, 1815

######## Genus *Delectopecten* Stewart, 1930

######### Delectopecten zacae

Taxon classificationAnimaliaPectinidaPectinidae

(Hertlein, 1935)

########## Examined material.

106 valves EA345 (2.5557°N, 79.0476°W) at 668 m (INV MOL9800).

########## New location.

Off Nariño, Colombian Pacific.

########## Distribution.

Mexico to Peru (Coan and Valentich-Scott 2012). Choco, Colombia (Gracia and Valentich-Scott 2014).

########## Remarks.

No live *Delectopectenzacae* specimens were collected during the present study. In northern Colombia (Choco), both live specimens and empty valves were found. The present finding extends the bathymetric range of this species to 668 m in the Colombian Pacific.

##### Order LIMIDA Moore, 1952

###### Superfamily LIMOIDEA Rafinesque, 1815

####### Family LIMIDAE Rafinesque, 1815

######## Genus *Limatula* Wood, 1839

######### Subgenus Limatula s.s. Wood, 1839

########## Limatula saturna

Taxon classificationAnimaliaLimidaLimidae

F.R. Bernard, 1978

Fig. 6

########### Examined material.

2 live specimens EA335 (1.7499°N, 79.5017°W) at 866 m (INV MOL9772).

########### New location.

Colombian Pacific.

########### Distribution.

U.S.A. to Mexico (Coan and Valentich-Scott 2012).

########### Remarks.

*Limatulasaturna* has been documented from Alaska to northern Mexico from 20–675 m (Coan and Valentich-Scott 2012).The Colombian specimens represent the first record for South America. Very recently (i.e., March 2018), this species has been observed in the region of Lambayeque, Peru (Valentich-Scott, pers. obs.).

##### Superorder HETEROCONCHIA J.E. Gray, 1854

###### Clade HETERODONTA Neumayr, 1884

####### Order LUCINIDA J.E. Gray, 1854

######## Superfamily LUCINOIDEA Fleming, 1828

######### Family LUCINIDAE Fleming, 1828

########## Genus *Lucinoma* Dall, 1901

########### Lucinoma heroica

Taxon classificationAnimaliaLucinidaLucinidae

(Dall, 1901)

Fig. 7

############ Examined material.

3 valves EA345 (2.5557°N, 79.0476°W) at 668 m (INV MOL9773).

############ New location.

Colombian Pacific.

############ Distribution.

Mexico to Peru (Coan and Valentich-Scott 2012).

############ Remarks.

*Lucinomaheroica* has previously been found in depths greater than 1,838 m (Coan and Valentich-Scott 2012). At 668 m, the Colombian specimens are the shallowest record for the species.

####### Order VENERIDA J.E. Gray, 1854

######## Superfamily GLOSSOIDEA J.E Gray, 1847

######### Family VESICOMYIDAE Dall & Simpson, 1901

########## Genus *Calyptogena* Dall, 1891

########### Calyptogena cf. gallardoi

Taxon classificationAnimaliaVeneridaVesicomyidae

Sellanes & Krylova, 2005

Fig. 8

############ Examined material.

1 valve EA345 (2.5557°N, 79.0476°W) at 668 m (INV MOL9805).

############ New location.

Off Nariño, Colombian Pacific.

############ Distribution.

South-central Chile, off Bahía de Concepción (Sellanes and Krylova 2005).

############ Remarks.

The single valve collected is insufficient to allow a definitive identification to species. The shape and dentition place it closest to *Calyptogenagallardoi*.

########## Genus *Pliocardia* Woodring, 1925

########### Pliocardia cf. donacia

Taxon classificationAnimaliaVeneridaVesicomyidae

(Dall, 1908)

############ Examined material.

1 valve EA337 (1.7811°N, 79.0351°W) at 530 m (INV MOL9768), 1 valve EA336 (1.9045°N, 79.3030°W) at 612 m (INV MOL9770), 1 live specimen EA344 (2.3905°N, 78.8288°W) at 656 m (INV MOL9771).

############ New location.

Off Nariño, Colombian Pacific.

############ Distribution.

Panama (Coan and Valentich-Scott 2012), Choco, Colombia (Gracia and Valentich-Scott 2014).

############ Remarks.

Prior to this study, dead shells of *Pliocardiadonacia* were identified in Pacific Colombia at depths between 272 and 295 m (Gracia and Valentich-Scott 2014). The present collection in southern Colombia yielded one live specimen and two empty valves, suggesting that the species inhabits both the northern and southern Colombian Pacific. Further, the bathymetric limit of the species is extended to 656 m in the Colombian Pacific. Many generic uncertainties exist within the family Vesicomyidae. Thus, we follow Coan and Valentich-Scott (2012) in their tentative placement of *P.donacia* within the genus *Pliocardia*.

####### Order MYOIDA Goldfuss, 1820

######## Suborder MYINA Goldfuss, 1820

######### Superfamily MYOIDEA Lamarck, 1809

########## Family CORBULIDAE Lamarck, 1818

########### Genus *Caryocorbula* J. Gardner, 1926

############ Carycorbula

Taxon classificationAnimaliaMyoidaCorbulidae

sp.

Fig. 9

############# Examined material.

1 valve EA331 (2.5078°N, 78.7993°W) at 350 m (INV MOL9763).

############# New location.

Off Nariño, Colombian Pacific.

############# Remarks.

This single valve from station EA331 is similar to several Panamic and Peru-Chile Province species of *Carycorbula*, but it is insufficient to allow a definitive identification to species.

######## Suborder SEPTIBRANCHIA Pelseneer, 1988

######### Superfamily CUSPIDARIOIDEA Dall, 1886

########## Family CUSPIDARIIDAE Dall, 1886

########## Genus *Cuspidaria* Nardo, 1840

########### Cuspidaria panamensis

Taxon classificationAnimaliaMyoidaCuspidariidae

Dall, 1908

Fig. 10

############ Examined material.

1 valve EA332 (1.6677°N, 79.1826°W) at 730 m (INV MOL9764), 7 valves EA345 (2.5557°N, 79.0476°W) at 668 m (INV MOL9765), 1 live specimen EA345 at 668 m (INV MOL9766), 4 live specimens A336 (1.9045°N, 79.3030°W) at 612 m (INV MOL9767).

############ New location.

Off Nariño, Colombian Pacific.

############ Distribution.

Panama (Coan & Valentich-Scott 2012).

############ Remarks.

*Cuspidariapanamensis* was previously known only been known from the type locality in the Gulf of Panama (Coan and Valentich-Scott 2012). Our records extend the distribution over 600 km to the south. Coan and Valentich-Scott (2012) indicate a maximum size of 41 mm for *Cuspidariapanamensis*. However, our material from station EA345 increases the maximum length to 44.2 mm.

######## Superfamily VERTICORDIOIDEA Stoliczka, 1870

######### Family VERTICORDIIDAE Stoliczka, 1870

########## Subfamily LYONSIELLINAE Dall, 1895

########### Genus *Dallicordia* Scarlato & Starobogatov, 1983

############ Dallicordia alaskana

Taxon classificationAnimaliaMyoidaVerticordiidae

(Dall, 1895)

Fig. 11

############# Examined material.

10 valves EA337 (1.7811°N, 79.0351°W) at 530 m (INV MOL9802), 14 live specimens EA337 at 530 m (INV MOL9803).

############# New location.

Off Nariño, Colombian Pacific.

############# Distribution.

Sitka, Alaska, to Tumbes, Peru (Coan and Valentich-Scott 2012).

############# Remarks.

This material represents a new record for the Colombian Pacific.

########### Genus *Lyonsiella* G. O. Sars, 1872

############ Lyonsiella cf. magnifica

Taxon classificationAnimaliaMyoidaVerticordiidae

Dall, 1913

Fig. 12

############# Examined material.

1 live specimen EA345 (2.5557°N, 79.0476°W) at 668 m (INV MOL9804).

############# New location.

Off Nariño, Colombian Pacific.

############# Distribution.

Mexico to Panama (Coan and Valentich-Scott 2012).

############# Remarks.

Colombian material resembles the type material of *Lyonsiellamagnifica.* However, our specimens have more prominent umbones, a more truncate anterior end, and a more obliquely truncate posterior end when compared to the type material. Many additional specimens would be necessary to determine if our single specimen falls within the range of intraspecific variation for *Lyonsiellamagnifica* or it represents a new species.

## Discussion

The new species of Malletiidae herein described brings to eight the number of known species for this family in the eastern Pacific Ocean (Table 3). *Malletia* is a widely distributed genus that is associated mainly with deep water and soft sediments (Coan and Valentich-Scott 2012). Previously reported from Colombia were *M.goniura* Dall, 1890 and *M.truncata* Dall, 1908 (type locality, Malpelo Island, at 3,334 m).

As was true of the previous study in the northern Colombian Pacific (Gracia and Valentich-Scott, 2014), these recent collections not only expand the geographic distributions of many species on the Colombian continental margin, but they also represent new collection locations. This serves as potential evidence for the species actually living in the area, rather than the transport of dead shells into the region. Further, our findings have significantly expanded the bathymetric limits of several species. One new species has been described, indicating that this region of Colombia is still relatively unexplored. Further surveys are necessary to complement this malacological inventory and to clarify the taxonomic identity of several species. These are important preliminary steps for to assist in investigating the impacts of anthropogenic practices and changes (e.g., deep-sea trawling, pollution).

Deep-sea baseline surveys seek to expand bivalve records for the Colombian Pacific Ocean. In 2014, Gracia and Valentich-Scott reported on specimens collected in the northern Colombian Pacific; 89.5% of the identified species represented new records for the region. The present survey used a similar methodology but was conducted in the southern Colombian Pacific. The number of bivalve species we encountered in the southern Colombian Pacific (16) was far lower than that for the northern Colombian Pacific. This could possibly be due to the different depths sampled in either survey, or possibly the decreasing diversity associated with increasing depth.

The transport of sediment caused by river discharge, marine currents, and other factors stimulate the resuspension of material on soft sediments (Segall et al. 1989). In the northern Colombian Pacific there is a greater influence of the equatorial countercurrent and the Panama Current, and the discharge from the Baudó River, while the southern Colombia Pacific (where Tumaco Bay is located) sees the influence of cold continental waters (CCCP 2002). All of these processes in Pacific Colombian result in a dense mixture of water and sediment that moves along the bottom of the sea and transports plant waste material. In both the northern (Choco) and the southern (Nariño) zones, a great abundance of sunken wood was encountered, indicating similar conditions influenced by terrestrial deposits.

Characteristics of sediments, currents, organic matter, availability of oxygen and many others factors could influence the composition, abundance, and occurrence of the benthic fauna. It should be noted that collections made in deep water in both northern and southern Pacific Colombia have yielded only a limited number of living bivalves and those that were numerically dominant were empty shells.

In conclusion, this paper serves as a contribution to our understanding of marine bivalves in deep waters of the southern Colombian Pacific. Our results reveal the importance of continued deep-sea research cruises in Colombia and subsequent taxonomic analysis of the specimens collected.

## Supplementary Material

XML Treatment for Ennucula panamina

XML Treatment for Nucula (Lamellinucula) iphigenia

XML Treatment for Jupiteria lobula

XML Treatment for Neilonella cf. atossa

XML Treatment for Malletia goniura

XML Treatment for Malletia tumaquensis

XML Treatment for Orthoyoldia panamensis

XML Treatment for Delectopecten zacae

XML Treatment for Limatula saturna

XML Treatment for Lucinoma heroica

XML Treatment for Calyptogena cf. gallardoi

XML Treatment for Pliocardia cf. donacia

XML Treatment for Carycorbula

XML Treatment for Cuspidaria panamensis

XML Treatment for Dallicordia alaskana

XML Treatment for Lyonsiella cf. magnifica
